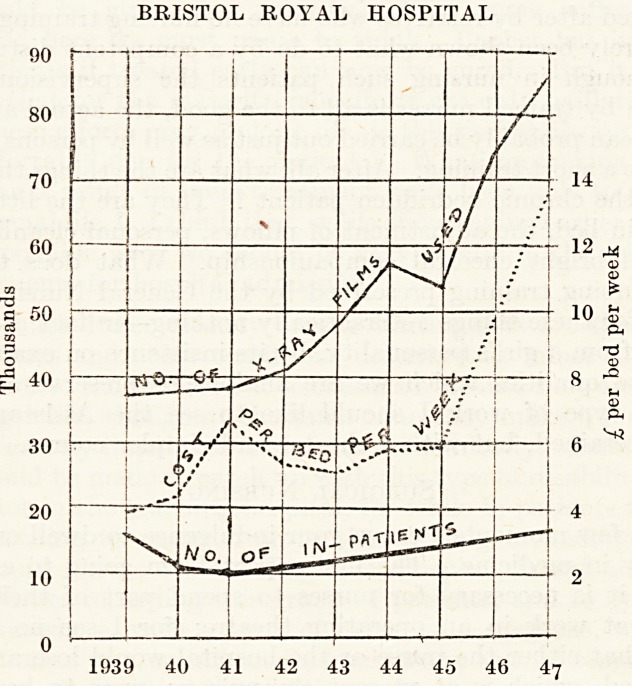# The Thirty-Sixth Long Fox Memorial Lecture: Some Aspects of Hospital Economy

**Published:** 1949-01

**Authors:** R. Milnes Walker

**Affiliations:** Professor of Surgery, University of Bristol


### THE THIRTY-SIXTH LONG FOX MEMORIAL LECTURE

BY

R. Milnes Walker, M.S.

*Professor of Surgery, University of Bristol*

DELIVERED IN THE UNIVERSITY OF BRISTOL ON TUESDAY, OCTOBER 19th, 1948,

ON

### SOME ASPECTS OF HOSPITAL ECONOMY

### Introduction

In 1894, when the British Medical Association held its Annual Meeting in this city, the Presidential Address was entitled: "On the Medical Man and the State." There is hardly a hint in that address of the commercial relations which now bind our profession to the State, there is no hint that up to that time the State had done anything for the medical profession, but most of the address dwells on what medicine had given to the State, the use of vaccination, the warnings about noxious trades, the benefits in public health brought about by the discoveries in bacteriology, and so on; the only reference to financial relations is in connection with the Poor-Law Medical Officer whose salary is described as so inadequate that the State ought never to sanction it; these posts are described as "ill-paid offices filled by men whose knowledge within the education of the present day leaves little to be desired, and whose influence for good excels, if possible, that of the rest of the profession, because exercised under greater difficulties". What would the speaker have to say now, when nearly all the general practitioners of the country, to whom the remarks apply equally well, have been coerced into a gigantic poor-law medical service?

Who was this speaker? None other than Edward Long Fox whose memory we are gathered together to honour to-day.

On looking through the titles of previous lectures I was surprised to find that there was not a single one which dealt with hospitals—and all the more surprised when considering Long Fox's life. For he must have had an intense interest in the organization and management of hospitals, being himself brought up in the atmosphere of the one at Brislington founded by his grandfather, and later serving on the active staff of the Bristol Royal Infirmary.

I would like to digress for a minute to give you my idea of a hospital; its original meaning was "a place of rest and entertainment", later "a charitable institution for the housing and maintenance of the needy, infirm or aged". Such was its use in the Middle Ages, when Bristol was so richly endowed with its Hospitals of St. Lawrence and St. Mary Magdalen for lepers, and of St. Mark, St. John the Baptist, St. Catherine, St. Bartholomew, and Holy Trinity. Incidentally it is worth noting that this last was the only one which was disclaimed by the Minister when the State took them over in 1539.

The subsequent period led to the popular idea of a hospital as defined by Sir Thomas Browne in *Religio Medici:* "For the World, I count it not an Inn, but an hospital, a place not to live in, but to dye in!" (Part II, Sect. 11.)

It was not until the eighteenth century, when so many of our well-known hospitals throughout the country were founded, that the present meaning really came into being. But to those of us who work in hospitals the idea is something different: the hospital is a team of living human beings working together for a common purpose, and given reasonable facilities to do this work, they need not necessarily be housed in marble palaces. We know of such magnificent buildings, where the latest gadgets are provided, where everything is spick and span, yet they hold no reputation as centres either of active research, of good teaching or of well-judged therapy; yet we are surprised very often at the humble places from which great things have come. One black sheep in such a team may mar the reputation of the whole for years, one misfit may disturb the smooth running of a complete hospital unit.

### THE NEED FOR HOSPITAL SERVICES

We have so far had no survey which gives us an indication of the need for hospital services; the nearest approach to it has just been published in the survey by the Nuffield Trust of the hospital-treated sickness in Stirlingshire. This gives us the amount of use which is made by the population of the county of the hospital services of this and surrounding districts. We find that in the course of a year one person in every seven made use of the hospital services, either as an in-patient or as an out-patient, while one in every seventeen was an in-patient with an average stay of about thirty days. If you were to take these figures as a basis of the need you would find that the South-Western Region is well supplied with hospital accommodation.

### COST OF HOSPITAL SERVICES

No thoughtful person can but be alarmed at the increasing cost of the hospital services. At the Bristol Royal Hospital the cost per patient per week has risen from under four pounds just before the war to over thirteen pounds now (see chart), and this will in future be considerably greater now that payment is being made for the services of the senior members of the medical staff which hitherto were given voluntarily. A certain amount of the increased cost is due to the great expansion of the ancillary methods of investigation, particularly radiology; but it might be profitable to review all departments with a critical eye. In fact every clinician who is responsible for the charge of patients in hospital should be constantly on his guard against the excessive use of any particular form of investigation or treatment, and must always employ the simple, straightforward methods when these will suffice.

BRISTOL ROYAL HOSPITAL

Another cause for alarm in the increasing cost of hospital services is the steadily rising age of the population. We cannot shut our eyes to the fact that infirmity and the need for hospital admission are greater in the aged than in the young. But there are a great many elderly people now occupying beds in infirmaries throughout the country, who with a little ingenuity and encouragement need not be bedridden, but can be up, and in many cases are capable of providing for their own domestic necessities. The results which have been achieved by Dr. Warren in Middlesex in this direction are remarkable, and no doubt can be repeated in other areas. The

County of Cornwall was already active in this matter before the introduction of the Health Service, and the Regional Hospital Board is taking active steps to face the problem.

### SHORTAGE OF NURSING STAFF

And this brings me to the immediate crux of the hospital problem, the shortage of nursing staff. In view of the changing age-groups of the population, I believe that we shall never return to the days when nursing labour will be plentiful, so we must adjust our plans accordingly. By this I do not mean that we should in any way slacken our efforts to recruit more girls to this profession, but that we must look at the problem from the other end as well. For example, we know that many of the chronic sick are exceedingly well looked after by relatives who have no nursing training, but who have merely been shown what to do by a competent district nurse. Thus, though in nursing such patients the supervision of their attention by trained nurses is all to the good, the actual attendance on them can probably be carried out just as well by persons who have had quite a short training. After all, what are the things that matter most to the chronic bedridden patient ? They are the little things, comfort in bed, the adjustment of pillows, personal cleanliness, and above all bright cheerful companionship. What does the three-years' nursing training prescribed by the General Nursing Council provide for these things ? Practically nothing—in fact, it probably detracts from a girl's personality, by its insistence on examinations, just those qualities which we are anxious to preserve and foster. For this type of work I should like to see the Assistant Nurses register retained, but with a shorter and simpler course.

### SURGICAL NURSING

For a few moments I crave your indulgence to dwell on my own speciality in medicine. The first aspect I am going to consider is whether it is necessary for nurses to spend part of their time in training at work in an operating theatre; for I see no reason to believe that either the nurse or the hospital would lose anything if this period, which is at present compulsory, were to be excluded from the training. The work in the operating theatres at present done by the nurses can be done by theatre technicians, male or female, specially trained for this purpose, who thus leave nurses to carry out the work for which they are primarily trained—nursing the sick.

There are many other ways by which we can relieve the work of a nurse in a surgical ward, for example, by getting patients out of bed and mobile as early as possible after operation, thus saving those duties for the bed-confined patient, connected with the excretory functions, which are naturally distasteful to patients and nurses alike.

### WAITING-LISTS

And now I must touch on the matter of waiting-lists. We have at the moment, to take a single example, on the general surgical waiting-list at the Bristol Royal Hospital, some 1,700 patients. Many of these will never be admitted ; by the time that their turn comes they may be dead, they may have moved elsewhere, or perhaps time alone will have healed them. Are we selecting the right patients for the beds which are available ? At the moment we give first priority to emergency admissions, accidents and acute illness, patients who are never on the waiting-list at all. Many of them are snatched from death or from prolonged serious disability and soon again return to their active place in the nation's life : it is right that they should be given first priority. Next come sufferers from cancer, and here we must pause to think. Cancer has its appeal. Many patients if treated early can now be cured. But it must be admitted that for the majority our efforts are only palliative : and though these efforts may prolong life, is this period always a happy and comfortable one for the patient ? What proportion of these patients as a result of their treatment have been able to return to active normal life ? I think that, while encouraging earlier diagnosis in every possible way, we should be very careful in the selection of cancer patients for hospital admission.

On a much lower priority on the waiting-lists are the patients with ruptures and other conditions described as minor ailments—not so minor to the sufferer but so classified because they are unlikely to endanger life, though at the same time they make life a misery, and may greatly impair his usefulness economically. Surely a great effort should be made to catch up with this type of disability. Their place is not in the teaching hospital, for while in-patients they contribute nothing to research or to the training of undergraduates. But I would like to see the Regional Hospital Board alive to its responsibilities regarding this group of patients, and to open a few wards somewhere, with a team of nurses and surgeons, who, as in an operation of war, would set themselves the task of removing this group from the waiting-lists. We might post a team to a small cottage hospital, and give all the patients with minor surgical disabilities who are on waiting-lists the opportunity of admission and early attendance to their case. The turnover would have to be rapid, and any patient who either required additional investigations or developed a complication demanding long-stay treatment would have to be transferred elsewhere. It would be interesting to know how many patients with ruptures or minor ailments of the rectum are on the waiting-lists of hospitals in the South-Western area and to what extent the failure to treat them without delay is influencing industrial efficiency.

Another aspect which deserves consideration is the provision of hostels in connection with our larger hospitals; many patients come from a distance either for special facilities in diagnosis or for some form of treatment which only requires a part of each day and does not necessitate their remaining in bed. A hostel for such patients would be most valuable and would relieve the in-patient beds.

### CONCLUSION

I have in this discourse only touched on some of the fringes of our hospital problems: but we cannot start with a clean sheet, and we must for the moment accept many things which are undesirable. However, that is no reason why we should not plan for the future and consider on what basis those plans should be made. An announcement has already been made of an inquiry sponsored by the Nuffield Trust, in co-operation with the University of Bristol, into the function and design of hospitals. It is not for me to attempt any conjecture as to what their findings will be, but that inquiry aims at being practical rather than Utopian, and it will have to take into consideration all the relevant facts. The one which to my mind stands out above all others is that in the future our labour resources are going to be scarce, particularly as regards nursing, and we shall have to make our plans with the incorporation of every labour-saving device, both in construction and equipment, if in the future we are going to make use of our hospitals when built. It is clear that there is a limit to the number of hospital beds which the nation can support, so our plans should be directed to making the most efficient use of these beds. There is nothing to be gained by adding to the number of beds if we cannot provide the labour to make use of them.

But there is the broader problem, as to who should be the inmates of our hospitals. I have already referred to the aged and chronic sick; if they do not go into institutions of some sort where else can they go? I am not going to speak of communal homes for old people, except to say that I consider it a pity to segregate old people from the other age-groups if this can be avoided. At the present time there are people who cannot be bothered to look after infirm relatives, either young or old: they wish to enjoy the pleasures of this world rather than accept its obligations. It has probably always been the same, but I am sure that the problem gets worse as the worldly distractions increase in number and intensity. Many old people are in institutions because their relatives have not the means, either financially or of accommodation, to look after them at home. But there are some who are there because their relatives do not *want* to look after them. The hospital services can only be relieved of the former by better housing and a better standard of living ; but they could be eased of the latter by a realization of the sense of family obligation. The report by Dr. J. H. Sheldon on Old Age in the Home shows how much is done for old people by their families, particularly amongst the poor, but I am sure that there is room for improvement which would help to relieve the problem of hospital accommodation.

However, we have got to face the fact that the cost of the hospital services is going to be a very heavy burden, and there are going to be some shocks when the figures become known, for the estimates made by the Ministry of Health are a long way short of the mark. But in the long run the limiting factor will not be finance, but labour. As bureaucracy absorbs an increasing proportion of the man-power, less and less remains for productive work. We have got to see that the administration of our hospital services is kept as small as is consistent with an efficient service, and that those trained in its technical aspects, particularly doctors and nurses, are not side-tracked from their useful and economical employment into the administrative machine.

Now that the State has taken over the responsibility of the hospital services, I cannot do better than quote from that presidential address which was delivered fifty-four years ago by Long Fox. " Happy is the State," said he, " that legislates in all things concerning the welfare of the people in dependence on medical advice. As a profession . . . we concern ourselves with the well-being of the masses of mankind, the possibilities of the advancement of the race, the annihilation of those things that militate against the progress of the nation. Our highest ambition is to leave each generation better than we found it, and by the results of our investigations and by the influence of our lives to form foundations for our successors to rise higher still, and to elevate the race to a point impossible for ourselves, to deserve to have it said of each one of us, as it was of the great head of our profession, ' He went about doing good, healing all that were diseased, for God was with him '." May the State take note of this advice, and may our profession continue to uphold these ideals in our new relationship with the hospitals.

## Figures and Tables

**Figure f1:**